# High-Quality Draft Single-Cell Genome Sequence Belonging to the Archaeal Candidate Division SA1, Isolated from Nereus Deep in the Red Sea

**DOI:** 10.1128/genomeA.00383-18

**Published:** 2018-05-10

**Authors:** David K. Ngugi, Ulrich Stingl

**Affiliations:** a Red Sea Research Center, King Abdullah University of Science and Technology, Thuwal, Saudi Arabia

## Abstract

Candidate division SA1 encompasses a phylogenetically coherent archaeal group ubiquitous in deep hypersaline anoxic brines around the globe. Recently, the genome sequences of two cultivated representatives from hypersaline soda lake sediments were published. Here, we present a single-cell genome sequence from Nereus Deep in the Red Sea that represents a putatively novel family within SA1.

## GENOME ANNOUNCEMENT

Environmental surveys of prokaryotic communities have revealed the existence of several novel uncultivated 16S rRNA gene lineages in deep hypersaline anoxic brines (DHABs [1]). Among these is the archaeal candidate division SA1, which branches basal to Haloarchaea (2). SA1 is believed to harbor clues to the evolutionary history of methanogenic and halophilic archaea (1). Recently, two methyl-reducing halophilic methanogenic strains from hypersaline soda lake sediments were discovered and proposed to be a new euryarchaeal class within SA1 called Methanonatronarchaeia (3), which provided first insights into the role of SA1 in DHABs.

These two SA1 strains diverge by up to 10% at the 16S rRNA gene level but affiliate with one of the two discrete phylogenetic clusters originally identified in the Shaban Deep brine pool in the Red Sea (2, 3). Here, we report a nearly complete single-cell genome sequence (that of SCG-AAA382-B04) of a novel SA1 clade member from Nereus Deep in the Red Sea.

Brine water samples were collected at a depth of 2,445 meters below sea level (mbsl) from Nereus Deep (23°11′53″ N, 37°25′09″ E) in November 2011. The sample site conditions were as follows: 30.1°C, 22.4% salinity, pH 5.5, and 2.8 µM dissolved oxygen (4). Single cells were sorted and amplified using the REPLI-g kit (Qiagen) at the Single Cell Genomics Center in the Bigelow Laboratory for Ocean Sciences, as described by Ngugi et al. (4).

A paired-end sequence library (2 × 101 bp) of the single-cell amplified genome (SAG) was prepared using the TruSeq DNA library kit and sequenced using an Illumina HiSeq sequencer at the Bioscience Core Laboratory at the King Abdullah University of Science and Technology (KAUST). Thirty-two million reads were quality trimmed using Trimmomatic version 0.32 (5) and assembled into contigs with SPAdes version 3.9.0 (6), applying the error correction and the single-cell mode. Genome completeness was estimated using CheckM (7).

The SAG is composed of 132 contigs totaling 1.42 Mbp (*N*_50_, 74.2 kbp; 720× coverage), with a G+C content of 35.5%. The SAG is of high quality, at 94.6% completeness (∼4.6% contamination), based on operational standards for SAGs (8), and contains 1,646 protein-coding genes plus 40 RNA-coding genes annotated with PGAP (9) and the Rapid Annotations using Subsystems Technology (RAST) server (10). The SAG contained a 16S rRNA gene sequence with 99% identity to environmental SA1 sequences from DHABs but only 93% identity to Methanonatronarchaeia representatives (3). Also, all three exhibit a relatively low degree of gene conservation (44 to 52%), syntenic orthologs (30 to 40%), and average amino acid identities (55 to 58%), suggesting that they likely encompass organisms from two families, considering the genomic threshold for this taxonomic rank (11, 12). The SAG has a proteome with a circumneutral isoelectric point and lacks osmolyte biosynthesis pathways, but it encodes potassium uptake systems, suggesting a reliance on potassium for osmoregulation. The methyl coenzyme M reductase complex implicated in methanogenesis (3) is absent, however, signifying the potential for a “nonmethanogenic” methylotrophic lifestyle for other SA1 lineages. We are currently reconstructing the full metabolism of these SA1 subclades.

### Accession number(s).

The whole-genome shotgun project has been deposited in GenBank under accession number PZKD00000000. The version described here is PZKD01000000.
